# Small-area spatio-temporal analyses of bladder and kidney cancer risk in Nova Scotia, Canada

**DOI:** 10.1186/s12889-016-2767-9

**Published:** 2016-02-19

**Authors:** Nathalie Saint-Jacques, Jonathan S. W. Lee, Patrick Brown, Jamie Stafford, Louise Parker, Trevor J. B. Dummer

**Affiliations:** Cancer Care Nova Scotia, Surveillance and Epidemiology Unit, Room 560 Bethune Building, 1276 South Street, Halifax, B3H 2Y9 NS Canada; Interdisciplinary PhD program, Dalhousie University, 6299 South Street, Room 314, PO Box 15000, Halifax, B3H 4R2 NS Canada; Department of Statistical Sciences, University of Toronto, 100 St. George St., Toronto, M5S 3G3 ON Canada; Cancer Care Ontario, 620 University Ave, Toronto, M5G 2 L7 ON Canada; Department of Pediatrics and Population Cancer Research Program, Dalhousie University, 1494 Carlton Street, PO Box 15000, Halifax, B3H 4R2 NS Canada; The University of British Columbia, School of Population and Public Health, 2206 East Mall, Vancouver, V6T 1Z3 BC Canada

**Keywords:** Small-area disease mapping, BYM model, Local-EM algorithm, Bladder and kidney cancer risk, Geostatistical analysis, Spatial autoregressive analyses

## Abstract

**Background:**

Bladder and kidney cancers are the ninth and twelfth most common type of cancer worldwide, respectively. Internationally, rates vary ten-fold, with several countries showing rising incidence. This study describes the spatial and spatio-temporal variations in the incidence risk of these diseases for Nova Scotia, a province located in Atlantic Canada, where rates for bladder and kidney cancer exceed those of the national average by about 25 % and 35 %, respectively.

**Methods:**

Cancer incidence in the 311 Communities of Nova-Scotia was analyzed with a spatial autoregressive model for the case counts of bladder and kidney cancers (3,232 and 2,143 total cases, respectively), accounting for each Community's population and including variables known to influence risk. A spatially-continuous analysis, using a geostatistical Local Expectation-Maximization smoothing algorithm, modeled finer-scale spatial variation in risk for south-western Nova Scotia (1,810 bladder and 957 kidney cases) and Cape Breton (1,101 bladder, 703 kidney).

**Results:**

Evidence of spatial variations in the risk of bladder and kidney cancer was demonstrated using both aggregated Community-level mapping and continuous-grid based localized mapping; and these were generally stable over time. The Community-level analysis suggested that much of this heterogeneity was not accounted for by known explanatory variables. There appears to be a north-east to south-west increasing gradient with a number of south-western Communities have risk of bladder or kidney cancer more than 10 % above the provincial average. Kidney cancer risk was also elevated in various northeastern communities. Over a 12 year period this exceedance translated in an excess of 200 cases. Patterns of variations in risk obtained from the spatially continuous smoothing analysis generally mirrored those from the Community-level autoregressive model, although these more localized risk estimates resulted in a larger spatial extent for which risk is likely to be elevated.

**Conclusions:**

Modelling the spatio-temporal distribution of disease risk enabled the quantification of risk relative to expected background levels and the identification of high risk areas. It also permitted the determination of the relative stability of the observed patterns over time and in this study, pointed to excess risk potentially driven by exposure to risk factors that act in a sustained manner over time.

**Electronic supplementary material:**

The online version of this article (doi:10.1186/s12889-016-2767-9) contains supplementary material, which is available to authorized users.

## Background

Urinary tract cancers comprise primarily cancers of the urinary bladder and kidney, the former accounting for approximately two-thirds of all cases diagnosed. Bladder cancer is the ninth most common type of cancer worldwide (~360,000 cases per year) and the 13^th^ most common cause of death from cancer (~145,000 deaths per year worldwide) [1, 2]. Kidney cancer is comparatively less common, ranking twelfth and accounting for an approximate 150,000 new cases and 78,000 deaths annually [3, 4].

Internationally, the incidence rates for bladder and kidney cancer have been reported to vary by as much as ten-fold between countries. Incidence tends to be higher in Southwestern Europe, North Africa (Egypt) and North America; and lower in South America and Asia [1, 4, 5]. Parkin [2] reports the highest estimated mortality rates to be in Egypt, where the world-standardized rate of 34 per 100,000 (in men) is more than three times higher than the highest rates in Europe (Denmark 10.4, Spain 9.7) and eight times that in the United States (US) (3.4).

Several countries show increasing incidence for both bladder and kidney cancers, although with evidence of some stabilization or even decreases during the 1990s [2, 4]. Recent trends in stage-specific incidence rates for bladder cancer in some US populations, suggest however, that rates may be stabilizing in late stage disease but continue to increase in noninvasive predominantly low grade disease [6]. Regardless of space, time or stage at diagnosis, rates are consistently higher for males than females [4, 5, 7–9]. In fact, in most developed countries, men are at least, a three to five time greater risk than women.

Past variations in the prevalence of known etiological factors, whether genetic, environmental, occupational or behavioural, may to some extent, contribute to the reported temporal and geographical variations of urinary tract cancers among populations worldwide. In addition, differences in the scope of case ascertainment between national cancer registries may result in some countries reporting solely invasive diagnoses while others may include non-invasive or in situ diseases. Some countries count only one primary cancer in subjects with multiple cancers in the urinary tract. In the Netherlands, such practice is thought to reduce the reported incidence of bladder cancer by up to 10 % [2]. Finally, variations in rates within and/or between countries can be partly driven by the introduction of new imaging techniques enabling the detection of pre-symptomatic tumours.

In Canada, bladder cancer incidence rates increased from 1970 to 1981 and have since gradually declined or stabilized [10–12]. Kidney cancer incidence rates have also stabilised in recent years among females, but continue to increase at a rate of about 1.3 % among males [10, 11, 13, 14]. Rates of both bladder and kidney cancer are particularly high in Nova Scotia (NS), a province of 900,000 people, in Atlantic Canada. NS consistently has some of the highest rates of cancer in Canada for both males and females and continues to show increases in the age-standardized incidence rates of both bladder and kidney cancers. For bladder cancer, age-adjusted incidence rates estimated for 2015 exceed those of the national average by about 25 and 30 % among males and females, respectively [11]. Similarly, for kidney cancer, excesses of 30 and 45 % have been reported among males and females, respectively. This noted excess burden of urinary tract malignancies in NS is unlikely to result from health system related factors (e.g. scope of case registration, imaging technology) given the relative uniformity of health care delivery within the country.

This study thus, describes spatial and spatio-temporal variations in the risk of bladder and kidney cancer for NS in order to identify those areas where rates are higher than what would be expected given the prevalence of known risk factors. This is an important step to guide both etiological research and public health interventions in the province. We use two geospatial methods for modelling disease risk, both of which are appropriate for low-density populations such as NS. The first approach is a Community-level analysis using a spatial autogregression (or Besag, York and Mollie model), a Bayesian method that models diseases risk for spatially aggregated case counts [15, 16]. The second approach estimates spatially continuous variation in risk using a Local Expectation Maximization (local-EM) smoothing algorithm, an emerging geostatistical method developed by Fan, Stafford and Brown [17], which models spatial and temporal variation in risk when cases are aggregated to time-varying spatial boundaries. To our knowledge, this is the first attempt to model the risk of bladder and kidney cancer in NS and one of the first epidemiological applications of the Local-EM algorithm for cancer mapping in Canada.

## Methods

### Data sources

#### Cancer incidence data

were obtained from the NS Cancer Registry and were divided into two cohorts: Cohort 1 included all NS residents diagnosed with bladder or kidney cancer between 1998 and 2010 and aged 20 years and older; Cohort 2 included cases diagnosed between 1980 and 2010 and aged 20 years and older. Cases were coded according to the International Classification of Diseases (ICD-O) as following: bladder (ICDO: 188.0-188.9; ICD-O-2/3: C67.0-C67.9); kidney (ICDO: 189.0; ICD-O-2/3: C64.9). Because of a change in disease-coding over time, bladder cases included both, in situ (36 %, period 1998–2010; 21 %, period 1980–2010; Table 1) and invasive diagnoses; kidney cases included invasive diagnoses only.

**Table 1:** Cases characteristics for the two periods under study, Nova Scotia, Canada

	Bladder			Kidney		
	Total	Females	Males	Total	Females	Males
**Period 1998 - 2010**						
*Nova Scotia*						
Cases diagnosed	3,292	834	2,458	2,199	863	1,336
Cases analyzed^*^	3,232	820	2,412	2,143	848	1,295
*In situ*	1,164	298	866	0	0	0
Invasive	2,068	522	1,546	2,143	848	1,295
Mean age at diagnosis (years)	71	71.2	70.5	65	66	63.7
Spatial referencing (%)						
Civic address	86.6	85.5	86.9	85.9	86.4	85.5
Postal code	2.29	2.07	2.36	2.10	1.65	2.39
Town name	11.1	12.4	10.7	12.0	11.9	12.1
**Period 1980 - 2010**						
*Nova Scotia*						
Cases diagnosed^†^	6,473	1,642	4,831	3,762	1,493	2,269
Mean age at diagnosis (years)	70	70.5	69.9	65	65.9	63.8
*South-western Nova Scotia*						
Cases analyzed	1,810	423	1,387	957	358	599
*In situ*	386	86	300	0	0	0
Invasive	1,424	337	1,087	957	358	599
Spatial referencing (%)						
Civic address	43.6	40.4	44.6	47.2	50.6	45.2
Postal code	52.9	56.3	51.9	49.8	46.1	52.1
Town name	3.4	3.3	3.5	2.9	3.3	2.7
*Cape Breton Island*						
Cases analyzed	1101	283	818	763	306	457
*In situ*	172	41	131	0	0	0
Invasive	929	242	687	763	306	457
Spatial referencing (%)						
Civic address	43.7	45.9	42.9	53.7	54.2	53.4
Postal code	47.0	41.3	48.9	39.2	36.6	40.9
Town name	9.4	12.7	8.2	7.1	9.2	5.7

*Excludes 116 cases (2.1 %) diagnosed in a Community for which population data was not available

^†^Excludes 21 bladder cases (0.32%) and 10 kidney cases (0.27%) due to unavailable spatial information

The Community-level (BYM) analysis was restricted to Cohort 1. This is because the proportion of cases with incomplete residential addresses (i.e. civic street address) was fairly large prior to 1998. During those early years, most cases were assigned to a town or a six-digit postal code, which vary greatly in size, especially between urban and rural settings. Depending on the spatial scale of analysis, one postal code may belong to several geographic units or one unit of geography may contain several postal codes, resulting in the potential misclassification of the spatially aggregated data. The spatially continuous-grid based (local-EM) analysis was able to accommodate data from the entire 30 year period (Cohort 2) because the method allows for both changes in the spatial distribution of risk over time, and accounts for uncertainties in location of cases where civic street addresses are missing but postal codes or administrative regions are known.

The Nova Scotia Civic Address File (NSCAF) was used to assign spatial locations (i.e. longitude-latitude coordinates) to all cases for which a civic street address was available. When civic address was unavailable, the Desktop Mapping Technologies Inc (DMTI) conversion file was used to geo-reference postal codes. For the Community-level model, where postal code was unavailable or located in rural areas, a gazetteer of place names was used to georeference the centroid of the town. For the spatially-continuous local-EM, where postal code was available, cases locations were treated as spatially censored somewhere within one of the census regions containing at least one address with the postal code in question. Where postal code was unavailable, the local-EM analysis used the Census Division boundaries as a second type of spatial censoring. Proportions of case by spatial data type, including the numbers of cases excluded from each analysis due to uncertainty in their spatial location, are shown in Table 1.

#### Population data

from seven census years (1981, 1986, 1991, 1996, 2001, 2006, and 2011) were used for this study. Each census provided counts of people aged 20 years and older by age and sex group, and were used as the denominator for cases diagnosed within two years of a given census period.

For the modelling of risk using the spatial autoregressive model, population estimates were aggregated at the Community level, a set of geographic administrative units, which represent groupings of neighbourhoods with a degree of shared identity and social processes [18]. This level of spatial aggregation represents the finest unit of geography for which boundaries are stable over time. There were 311 Communities in NS over the study period with population counts up to 30,900 persons. In total, 36 Communities (30 First Nations Communities and 6 wilderness and park Communities) were excluded due to unavailable population information.

The spatially-continuous (local EM) analysis used population counts by age and sex group at the finest level of geography for which digitized spatial boundary data were available. These were census subdivision level (CSD) for the 1981 and 1986 census years; enumeration areas (EA) for the 1991 and 1996 census years; and dissemination areas (DA) for census 2001 onward. There were 113 CSD in 1981 and 118 CSD in 1986. The number of EA/DA ranged from 1379 to 1645 between the 1991 and 2011 census periods; their size varied to target a population of 400 to 700 individuals.

It was assumed that populations were uniformly distributed within these finest levels of census regions, a not unreasonable assumption if one accepts that these census regions generally follow physical boundaries, such as major streets and waterways, and are designed to be fairly homogeneous. An exception is regions which are indicated by Statistics Canada to be partially uninhabited, or lying outside the population ecumene, in which case the population is assumed to be homogeneously distributed within the inhabited portion.

#### Covariates

included in the Community-level spatial autoregressive model were indicators of socioeconomic deprivation and well water usage. The latter obtained from NS Environment, aimed to account for spatial variations in risk which may relate to exposure to environmental sources of heavy metals such as arsenic in drinking water, a known risk factor for the development of bladder and kidney cancer [19]. Socioeconomic deprivation indicators were derived from socio-economic data obtained from Statistics Canada. They were constructed as Community-level area-based composite indices of social and material deprivation intended to be used as a proxy for unavailable individual-level measures such as smoking, a key factor in the development of urinary tract malignancies. Material and social deprivations indices were also used to capture the contextual setting of a place of residence, which has been shown to independently predict smoking habit in both men and women and other health outcomes [20-24]. Each index summarized information relating to six socioeconomic indicators from the 2006 Canadian Census; all of which having known links to health outcomes and known application as geographic proxies of socioeconomic conditions [21, 25-28]. For people age 15 years and over, these variables were: the proportion of people with no high school diploma, the individual average income, the employment rate, the proportion of separated, divorced or widowed, the proportion of single-parent families, and the proportion of persons living alone. The first three indicators reflect the material dimension of deprivation; the others reflect its social aspect. Variables were combined using a Principal Component Analysis (PCA), a standard factorial approach that recognizes the interlinked nature of variables by accounting for their correlation and co-variation [29]. Methodological details appear in Saint-Jacques et al. [30]. Covariates were not included in the spatially-continuous analysis as the local-EM method does not currently accommodate covariates.

### Data analyses

#### Community-level analysis

The Besag York and Mollié (BYM) model (see [15, 16]), a popular and convenient spatial autoregressive model for count data referenced to discrete spatial regions, was used to perform Community-level analysis. The approach treats the case counts by Community as response variables, rather than Standardized Incidence Ratios (SIR), because the latter is unstable when computed from low counts. This is particularly important in this study due to the low population density of NS and the rarity of the health outcomes measured. Possible spatial dependence in the data, with pairs of nearby Communities tending to be more similar than Communities situated far apart, is accounted for with the inclusion of a spatially autocorrelated random effect term. The BYM models the case counts as Poisson distributed and supports Baysesian inference for model fitting, which in this study, was performed separately for each data set (bladder male, bladder female; kidney male, kidney female) using Integrated Nested Laplace Approximations [31]. Further details pertaining to this analytical approach are described in Additional file 1.

#### Spatially-continuous analysis

The local-EM kernel smoothing was used to perform the spatially-continuous analysis. The method developed by Fan, Stafford and Brown [17] was extended by Lee et al. (Lee J, Nguyen P, Brown P, Stafford J, Saint-Jacques N: Local-EM Algorithm for Spatio-Temporal Analysis with application in Southwestern Nova Scotia. Submitted in *Ann Appl Stat;* [32]) to accommodate the requirements of modelling the cancer incidence data presented here. Collected between 1980 and 2010, the data were subject to aggregation boundaries changing over time and were geocoded with varying degrees of precision. Exact spatial locations were derived from full residential civic street addresses for most of the recent cancer cases, though the proportion of cases spatially referenced with partial street address (i.e. postal codes) or with census regions, increased with the age of the data. Where exact location is unavailable, the local-EM kernel smoothing algorithm produces an optimal risk surface which averages out all the possible locations at which each case could be located. The bandwidth of the smoothing kernel is chosen by cross-validation (see Additional files 2 and 3) and determines the degree of smoothing in the risk surfaces. A detailed description of the methodology is contained in Lee et al. (Lee J, Nguyen P, Brown P, Stafford J, Saint-Jacques N: Local-EM Algorithm for Spatio-Temporal Analysis with application in Southwestern Nova Scotia. Submitted in Ann Appl Stat) and in Nguyen et al. [32], and summarized in Additional file 1.

In this study, local-EM analyses focused on two regions of the province which the BYM models suggested risk was particularly high, as to describe localized patterns in risk. Two models were applied: (1) a spatial model testing for significant variation in risk over space, and where a spatial effect was detected; (2) a spatio-temporal model was applied to determine whether risk also varied significantly over time. Maps were produced where statistically significant spatial or spatio-temporal effects were detected. Estimated risk surfaces based on local-EM are not presented to minimize risk of disclosure of personal health information. Rather, a p-value for testing for relative risk being lower than 1.1 (risk less than 10 % above the population average) at each location and time is presented. These p-values were computed with a parametric bootstrap, with 100 synthetic datasets simulated with a constant relative risk of λ(*s*,*t*) = 1.1 and for each *s* and *t* the p-value is the proportion of these datasets where the local-EM algorithm yields risk estimates exceeding the estimate produced by the data. Shown are exceedance probabilities, or one minus the p-values, which are large when risk is believed to exceed 1.1.

The software used was R version 3.1.1 (http://www.r-project.org) in combination with the *disease mapping* package [33] and the INLA software [34]. This study received ethics approval from Capital Health Research Ethics Board. The study was a secondary analysis of anonymised cancer registry data obtained from the NS Provincial Cancer Registry and a waiver of consent was approved.

## Results

### Cohort characteristics summary

A total of 6,473 bladder cancers and 3,762 kidney cancers were diagnosed in NS between 1980 and 2010 (Table 1), 95 % of which included spatial information on residence at time of diagnosis and were successfully geo-referenced. In total, 3,232 bladder and 2,143 kidney cancers were included in the analyses focusing on the 1998–2010 time period, and; 2,911 bladder and 1,720 kidney cancers were included in the analyses covering the 1980–2010 time period, which focused specifically on cases diagnosed in south-western (SW) NS (2,767 cases) and Cape Breton (CB; 1,864 cases) — two regions where risk was mapped at a finer spatial resolution. Geo-referencing based on exact residential location at diagnosis was more common for cases diagnosed in the most recent time period, between 1998 and 2010 (bladder 86.6 %; kidney 85.9 %) than for cases diagnosed between 1980 and 2010 (SW: bladder 43.6 %; kidney 47.2 %; CB: bladder 43.7 %; kidney 53.7 %). On average, kidney malignancies were diagnosed at a slightly younger age than bladder cancers (65 vs 70 years). Overall, the male to female ratio was about 2.9 and 1.5 for bladder and kidney cancer diagnoses, respectively.

### Spatial patterns of bladder cancer

#### Community-level analysis

Estimates and credible intervals for regression and variance parameters obtained from the BYM models are shown in Table 2. These coefficients represent the log relative risk in bladder cancer incidence over the entire province and study period. None of the covariates – well water usage or material and social deprivation – significantly affected the estimated risk for bladder cancer among males and females (Table 2). Thus, much of the observed spatial heterogeneity in risk relates to unmeasured risk factors which appeared to have a similar effect on the distribution of disease in both males and females. Both the spatially correlated and the independent random errors have standard deviations in the range of 0.1 to 0.4, reasonably large values considering that they apply to risk on the log scale (Table 2).

**Table 2:** Posterior summaries for regression and variance parameters – Bladder cancer, Nova Scotia 1998-2010

Bladder cancer	Males			Females		
Parameter	Mean	2.5 %	97.5 %	Mean	2.5 %	97.5 %
Intercept	−0.105	−0.297	0.086	0.007	−0.301	0.309
% using well water	0.001	−0.002	0.003	−0.001	−0.005	0.003
Material deprivation	−0.297	−0.109	0.048	0.055	−0.067	0.178
Social deprivation	0.046	−0.023	0.116	−0.018	−0.130	0.094
Spatial standard deviation	0.228	0.157	0.352	0.199	0.086	0.439
Unstructured standard deviation	0.124	0.072	0.193	0.240	0.126	0.421

**Fig. 1:**
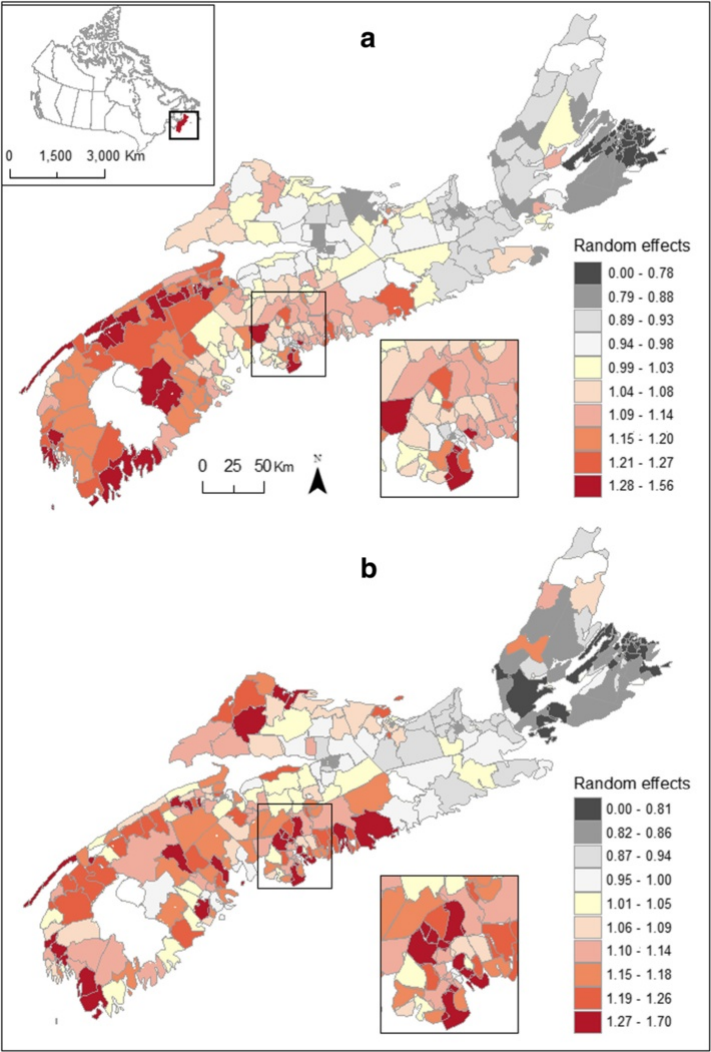
Posterior means relative risks for male (**a**) and female (**b**) bladder cancer, Nova Scotia 1998–2010

Figure 1 maps the residual spatial variation in bladder cancer risk, more specifically the posterior means E[exp(*U*_*i*_)|data] of the exponentiated random effects, among males (Fig. 1a) and females (Fig. 1b). These values are equivalently the ratio between the predicted risk λ_*i*_ for each community and the risk exp(μ + *X*_*i*_β) which is typical given the region's covariates *X*_*i*_. Regions of elevated risk are common in the south-western section of the province where several communities exhibit risk well above what is typical (i.e. > 1.2). Looking at these Community-level variations for the province, one identifies a clear southwest to northeast gradient among females, additional pockets of high risk being observed in Cumberland county (north central region).

**Fig. 2:**
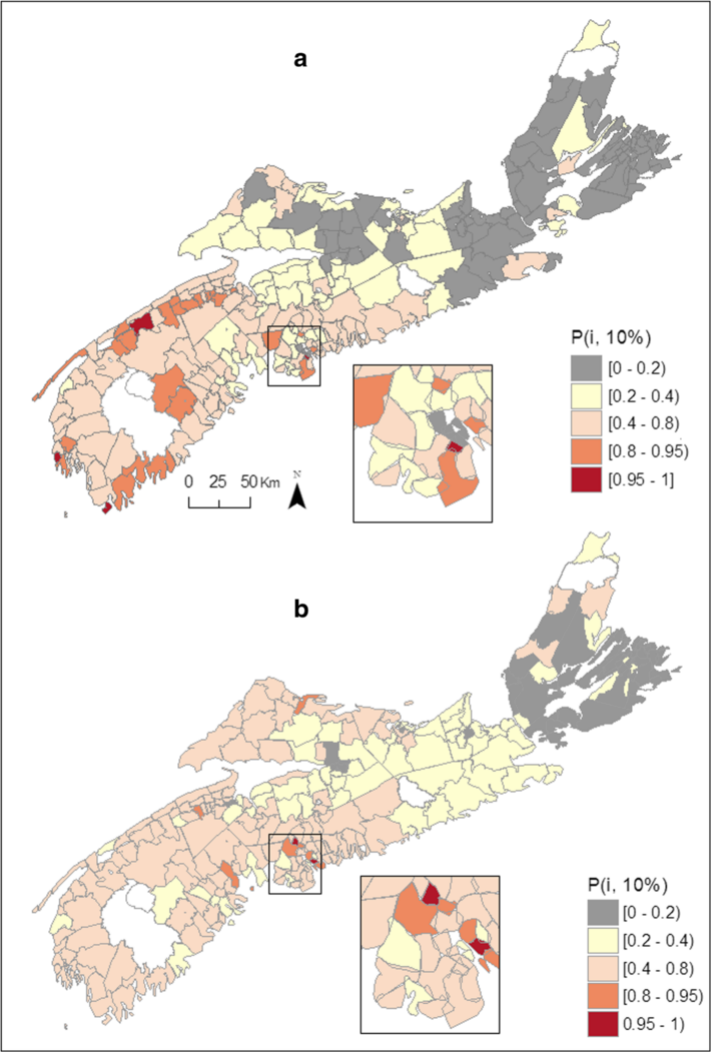
Exceedance probabilities (*P*_*i*_(10 %)) for male (**a**) and female (**b**) bladder cancer, Nova Scotia 1998–2010

Uncertainties associated with these maps can be visualized with exceedance probabilities, which are the probabilities that the risk in a Community or location exceeds a given threshold, defined here as 10 % above the risk that would be typical given the region's deprivation and well water usage. We denote these probabilities as *P*_*i*_(10 %) = *Pr*{λ_*i*_ > [1.1 exp(μ + *X*_*i*_β)] | data}, or equivalently *Pr*[exp(*U*_*i*_) >1.1|data]. Figure 2a shows exceedance probabilities for bladder cancer amongst males, with 28 communities in SW NS having a probability *P*_*i*_(10 %) in excess of 80 % and four communities having *P*_*i*_(10 %) >95 %, again supporting a southwest to northeast gradient. Estimated risk in these communities ranged between 1.24 –1.56, and between 1.39 – 1.56, respectively. The exceedance probabilities for females in SW NS are for the most part in the range of 0.2 – 0.8 (Fig. 2b), as the smaller number of cases for female cancers makes it more difficult to assess with any certainty whether a region has risk above or below a given threshold. In total of 9 Communities show exceedance probabilities for female risk above 80 % and 2 have probabilities above 95 %, the latter located in south central NS (Fig. 2b). Risk in those areas was higher than that estimated for males, with risk ranging between 1.38 – 1.69 and between 1.58 –1.69, respectively. Over the 12 year-period, high risk areas (*Pr*[exp(*U*_*i*_) >1.1|data] > 80 %) had 33 and 52 % more cases of male and female bladder cancer being diagnosed, respectively.

#### Spatially-continuous analysis

Table 3 a shows optimal spatial and spatio-temporal bandwidths obtained from cross-validation scores (Additional files 2 and 3) and p-values of Scores-Test that assess the statistical significance for spatial and spatio-temporal effects in bladder cancer risk in SW NS and CB. Spatial and spatio-temporal bandwidths determine the extent of the smoothing kernel used in risk estimation, and in this study, they ranged between 3 km and 22 km in space and 5 to 13 years over time. Based on these bandwidths, we observed significant localized variations in the spatial distribution of bladder cancer risk for males from both SW NS and CB regions (Table 3). For SW NS, the results suggested that these spatial patterns also varied over time (Table 3; p = 0.07). Statistically significant spatial variations in bladder cancer risk were not observed in females from either SW NS or CB regions (Table 3). These results possibly reflect a combination of small case counts and location misclassification. For example, there were only 247 cases of female bladder diagnosed between 1980 and 2010 in Cape Breton, and 76 % of those were geocoded to a single location. During cross validation, half the cases would be excluded from model fitting and optimal spatial bandwidths would be determined based on too few events to produce stable and statistically significant results.

**Table 3:** Optimal spatial and temporal bandwidth (BW) from cross-validation scores, bladder and kidney cancer, Nova Scotia 1980-2010

		Spatial		Spatio-temporal		
Region	Sex	BW (Km)	P-value	BW (Km)	BW (years)	P-value
Bladder						
SW	M	11	<0.001	11	13	0.07
	F	3	0.41	-	-	-
CB	M	4	0.01	4	13	>0.2
	F	22	0.79	-	-	-
Kidney						
SW	M	3	0.03	3	17	>0.2
	F	7	0.05	7	13	>0.2
CB	M	6	0.01	6	5	>0.2
	F	10	0.38	-	-	-

**Fig. 3:**
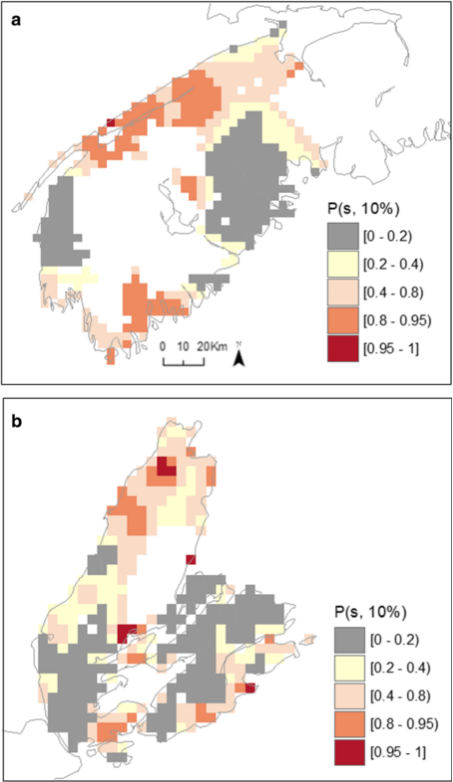
Bootstrapped exceedance probabilities (*P*(*s*; 10 %)) for risk surface of male bladder cancer in south-western Nova Scotia (**a**) and Cape Breton (**b**) regions

Exceedance probabilities obtained from fitting a spatially continuous risk surface with the local-EM algorithm are shown in Fig. 3 for male bladder cancer in SW NS and CB. These exceedance probabilities can be interpreted in a similar manner to the quantities from the BYM model shown in Fig. 2, with one difference being they refer to a threshold of 10 % above the average risk for NS without adjustment for deprivation and well water usage. Another difference is these probabilities vary over a continuous spatial surface as opposed to between Communities with set boundaries and, hence, provide insights on finer resolution patterns in risk. Thus, we write, *P(s;10 %)* as one minus a p-value for testing λ*(s)* < 1.1 with probabilities being computed using parametric bootstrapping (see details in Nguyen et al. [32] and Lee et al. (Lee J, Nguyen P, Brown P, Stafford J, Saint-Jacques N: Local-EM Algorithm for Spatio-Temporal Analysis with application in Southwestern Nova Scotia. Submitted in Ann Appl Stat). As observed using Bayesian inference, results from these finer-scale analyses also show probabilities of above-average risk in excess of 80 % along the Fundy shore and near Cape Sable Island and Shelburne, areas located on the south shore of NS (Fig. 3a). In Cape Breton, patterns of exceedance probabilities in excess of 80 % (Fig. 3b) pointed to areas of elevated risk where aggregated analysis based on BYM modeling had shown *P*_*i*_(10 %) to be less than 20 % (Fig. 2a).

**Fig. 4:**
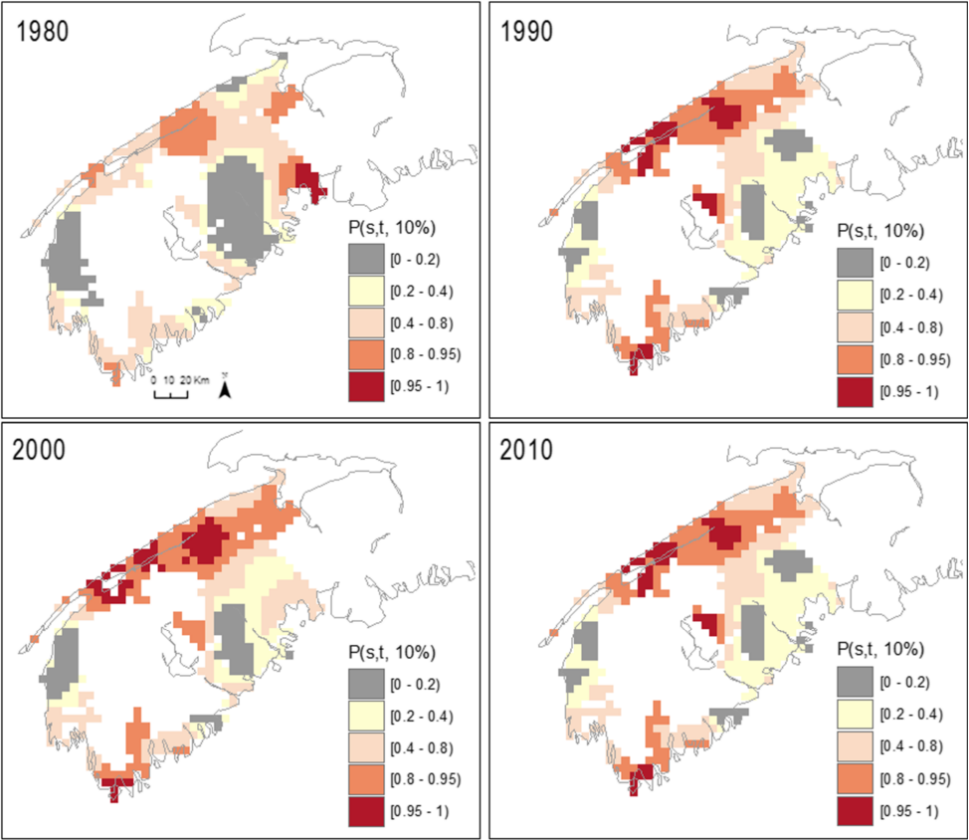
Bootstrapped exceedance probabilities (*P*(*s, t*; 10 %)) for risk surface of male bladder cancer for 1980, 1990, 2000, 2010, in south-western Nova Scotia

Figure 4 shows the exceedance probabilities obtained from fitting a spatio-temporal risk surface to male bladder cancer for SW NS, a region where risk varied over time (Table 3). In this latter model, where risk varies in time as well as in space, we write *P(s,t;10 %)* as one minus a p-value for testing λ*(s,t)* < 1.1. Here, *P(s,t;10 %)* is shown for four specific years, 1980, 1990, 2000 and 2010. Exceedance probabilities for the intervening years can be found in the supplementary materials and at http://pbrown.ca/jlee/spatio_temporal/. Note that while patterns of exceedance probabilities for year 2000 (i.e. Fig. 4c) includes data from 1980–2010, the 13 years closest to this index year will have the greatest influence upon parameter estimates. This is because the relative influence is determined by a weighting function that follows a Gaussian distribution with a standard deviation of 13 years (i.e. optimal temporal bandwidth for male bladder cancer). Simultaneously, the spatial weighting function associated with a point estimate also follows from a Gaussian distribution with a standard deviation of 11 km (i.e. optimal spatial bandwidth for male bladder cancer). Overall, the results are similar to those obtained with the spatial model, highlighting large areas with *P(s,t;10 %)* above 80 % along the Fundy Shore and south portion of the region. However, when adding a temporal component and thus further zooming into a finer scale of analyses, several locations show *P(s,t; 10 %)* surpassing 95 %, pointing to broad areas of significantly elevated risk where the estimated relative risk varied between 1.27 – 2.84 (not shown).

### Spatial patterns of kidney cancer

#### Community-level analysis

**Fig. 5:**
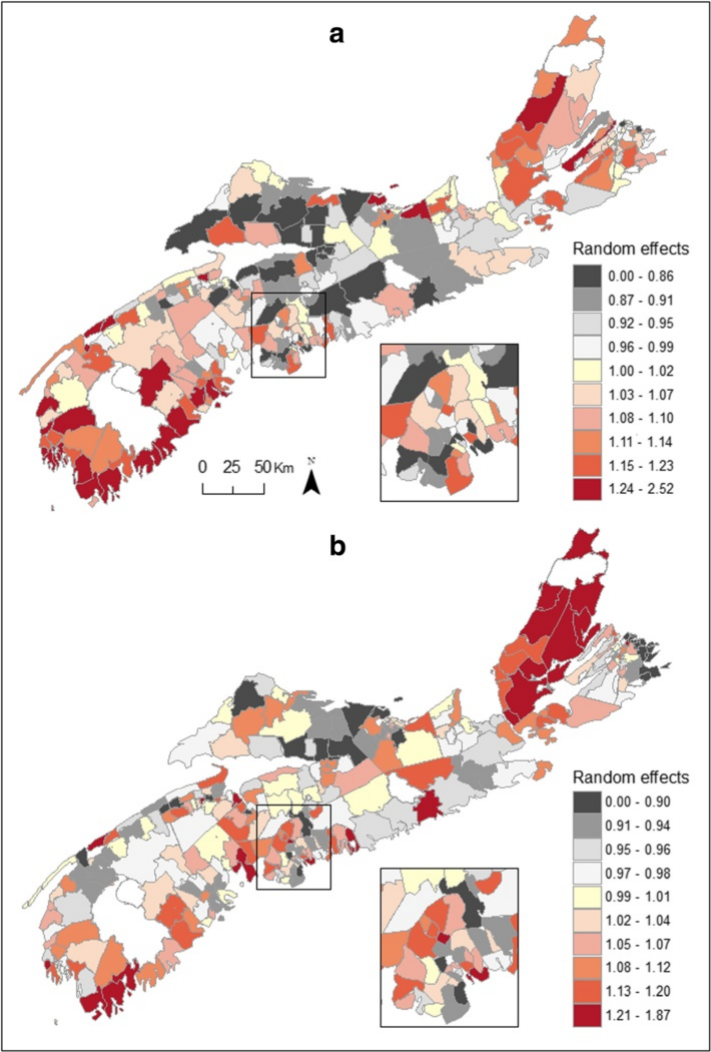
Posterior means relative risks for male (**a**) and female (**b**) kidney cancer, Nova Scotia 1998–2010

**Fig. 6:**
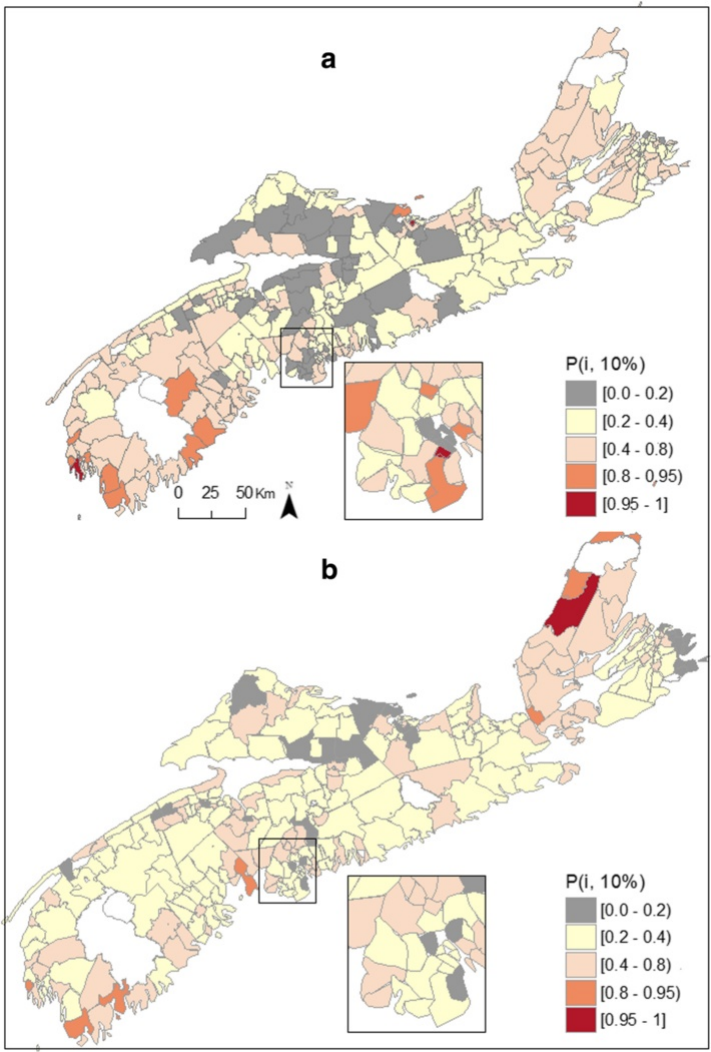
Exceedance probabilities (*P*_*i*_(10 %)) for male (**a**) and female (**b**) kidney cancer, Nova Scotia 1998–2010

As observed for bladder cancer, posterior summaries for regression and variance parameters show that the measured covariates had no significant influence on the estimated risk of kidney cancer (Table 4). Random effects for both spatially and unstructured random errors were significant, although showing greater unstructured heterogeneity for males than previously observed with male bladder cancer risk (i.e. ranging between 0.17 – 0.27 *vs* 0.07 – 0.19, respectively; Tables 2, 4). Maps of posterior means displayed strong spatial heterogeneity in male and female kidney cancer risk (Fig. 5a-b). Regions of elevated risk for male kidney cancer were common in the south-western region of the province as well as in several communities of CB Island, correlating with the elevated risk observed amongst females which is uniformly high in that region (Fig. 5a-b). Female kidney cancer rates were elevated in some Communities along the southern shore of SW NS and around the south shore of central NS (Fig. 5b). Figure 6a-b shows *P*_*i*_(10 %) for kidney cancer and a risk threshold that would be typical given the region's deprivation and well water usage. In total, 11 Communities showed *P*_*i*_(10 %) in excess of 80 % amongst males (estimated risk: 1.36 – 2.52); 2 of these being statistically significant (i.e. *Pr*[exp(*U*_*i*_) >1.1|data) >0.95; estimated risk: 1.73 – 2.52). The majority of these Communities are located along the south shore of SW NS (Fig. 6a). Exceedance probabilities above 80 % for females risk were observed in 8 Communities (estimated risk: 1.35 – 1.86); 4 located along the south shore of SW NS and 4 along the north shore of CB (Fig. 6b). Of these, 1 had a statistically significant probability (estimated risk: 1.87). Over the 12 year-period, high risk areas (*Pr*[exp(*U*_*i*_) >1.1|data] > 80 %) had 52 and 57 % more cases of male and female kidney cancer being diagnosed, respectively.

**Table 4:** Posterior summaries for regression and variance parameters – Kidney cancer, Nova Scotia 1998-2010

Kidney cancer	Males			Females		
Parameter	Mean	2.5 %	97.5 %	Mean	2.5 %	97.5 %
Intercept	0.032	−0.231	0.290	0.038	−0.259	0.326
% using well water	−0.001	−0.004	0.002	−0.001	−0.004	0.003
Material deprivation	−0.006	−0.112	0.097	0.052	−0.064	0.167
Social deprivation	0.008	−0.087	0.103	0.0004	−0.107	0.109
Spatial standard deviation	0.138	0.048	0.298	0.156	0.052	0.366
Unstructured standard deviation	0.265	0.174	0.390	0.251	0.137	0.440

#### Spatially continuous analysis

Optimal spatial and spatio-temporal bandwidths from cross-validation scores (Additional files 2 and 3) and associated p-values testing for spatial and spatio-temporal effects in kidney cancer risk, are shown in Table 3. Based on these bandwidths, we observed significant variation in the spatial distribution of kidney cancer risk in males and females from SW NS and in males from CB. Statistically significant spatio-temporal effects were not observed (Table 3; p > 0.2) and therefore maps of exceedance probabilities were derived from the spatial models with 30 years of pooled data (1980–2010). In comparison to the results obtained with BYM modeling, probabilities in excess of 80 and 95 % had a larger spatial extent. This pattern was generally observed across regions and genders. In addition, the probabilities produced by local-EM were less spatially smooth, allowing the detection of more localized risk. Again, *P(s;10 %)* for males in SW NS showed a high probability of excess risk along the southern shore, but also toward the centre of the region. Significant probabilities of exceedance in risk of male kidney cancer were also detected in several areas of CB; an occurrence that was not observed with BYM models (Fig. 6a, 7b). Correspondingly, exceedance probabilities for females were high along the southern shore of SW NS (Fig. 8). Overall, estimated relative risk for female kidney cancer ranged between 1.34 – 1.98 and 1.45 –1.98, for *P(s;10 %)*|data) > 0.80 and *P(s;10 %)*|data) > 0.95, respectively. For males, these values ranged between 1.53 – 2.54 and 2.01 –2.54.

**Fig. 7:**
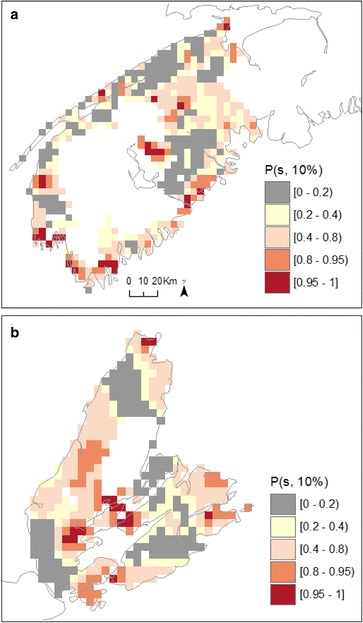
Bootstrapped exceedance probabilities (*P*(*s*; 10 %)) for risk surface of male kidney cancer in south-western Nova Scotia (**a**) and Cape Breton (**b**) regions

**Fig. 8:**
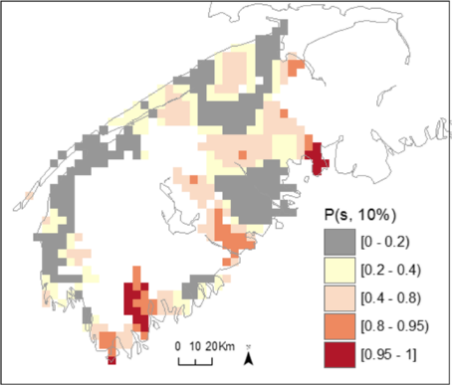
Bootstrapped exceedance probabilities (*P*(*s*; 10 %)) for risk surface of female kidney cancer in south-western Nova Scotia

## Discussion

### Summary of findings

This study showed evidence of spatial variation in the risk of bladder and kidney cancer in Nova Scotia. Posterior summaries for regression and variance parameters suggested that much of the heterogeneity in risk related to unmeasured risk factors. High risk areas for bladder cancer were predominantly distributed along a southwest to northeast gradient. Kidney cancer risk followed a similar distribution, although areas of elevated risk were also detected in various northeast Communities of Cape Breton, for both genders. Focusing on aggregated spatial units (Communities), the study showed that areas identified to have high probability of exceedance (BYM: *Pr*[exp(*U*_*i*_) >1.1|data] > 80 %) in the risk of male (28 Communities) or female (9 Communities) bladder cancer had 33 % (males) and 52 % (females) more cases diagnosed over the 12 year period, compared to the number of cases expected. Similarly, high risk areas for male (11 Communities) or female (8 Communities) kidney cancer had 52 % (males) and 57 % (females) more cases diagnosed than expected. From a public health perspective, this translates in an excess of nearly 200 urinary tract cancer (UTC) cases (150 bladder; 45 kidney) being diagnosed in those high risk areas where the estimated risk was observed to be at least 10 % above the NS average rate. Over a 12 year period, this corresponds to an additional 16 UTC cases annually, a conservative figure given that exceedance probabilities in excess of both 80 % and 95 % had much larger spatial extent when derived from the spatially-continuous analysis than with the Community-level model. This was true for risk measured in either sex or cancer site. Focusing on localized spatial patterns, this study also highlighted significant spatial and spatio-temporal variations in the risk of male bladder cancer within SW NS, with areas of elevated risk along the Fundy shore and south shore of the region. Elevated risk of both, male and female kidney cancer were also observed along the south shore of SW NS. In addition, risk for both male bladder and kidney cancer varied significantly in CB, although areas of elevated risk did not always overlap. Overall, spatial patterns were generally stable over time.

### Interpretation of spatial patterns

Patterns of spatiotemporal heterogeneity in risk provide clues to the occurrence and influence of extrinsic factors involved in the rise or fall of a disease. In this study, patterns of spatial variations in bladder and kidney cancers risk were stable over time, suggesting persistent risk exposure. The exception being male bladder, for which the results pointed to a temporal effect. However, the pattern of spatial variations in risk remained stable over a 13 year period, possibly also reflecting persistent effects. Similarly, a study of space-time patterns of bladder cancer incidence in Utah, US, detected high risk areas that were persistent over time [35]. These high relative risk areas were subsequently found to be associated with the presence of Toxic Release Inventory sites, where the risk was observed to range between 1.14 and 1.82 for both genders combined and between 1.12 to 1.47 for males only. While the processes generating the elevated risk in NS are unknown, the magnitude of the estimated risk in high risk areas for NS was similar to that reported in Utah, ranging between 1.24 – 1.56 and 1.38 – 1.69 among males and females, respectively based on BYM and between 1.48 – 1.99 and 1.48 – 1.95 among male from SW NS and CB, respectively, when based on local-EM. The latter tighter lower bounds of the estimates are attributable to the more conservative rule of exceedance probability applied in NS (NS: *P*_*i*_(10 %) > 0.8 and *P(s;10 %)* > 0.8; Utah: *P*(exp(*s*_*i*_) >1.0|data) > 0.8) for the determination of high risk areas. Both studies suggest an increased effect in females.

Several factors affect the incidence of urinary tract cancers worldwide. Exposure to tobacco smoke, occupational toxins and environmental source of heavy metals such as arsenic in drinking water, are amongst well established risk factors for bladder cancer, in particular, transitional cell carcinoma which account for 90 % of the bladder cancer cases diagnosed in developed countries [5, 7, 19]. Tobacco smoking [5, 9, 36–41] and long-term exposure to high levels of arsenic in drinking water also increase kidney cancer risk [19, 42] along with obesity [38, 43, 44], hypertension [38], the use of phenacitin-containing analgesics and exposure to trichloroethylene and polycyclic aromatic hydrocarbons [38, 45–47]. Whether measured independently or synergistically, the magnitude of influence of these risk factors for the development of UTC varies. However, meta-analyses of over 30 years of epidemiological studies suggest, for instance, that tobacco smoking could increase the risk of bladder and kidney cancer by at least 270 and 50 %, respectively, in current smokers compared to non-smokers [37, 48]. Exposure to arsenic in drinking water shows effects of similar magnitude, increasing the risk of bladder cancer by about 40 %, 230 and 310 % at levels exposure of 10, 50 and 150 μg/L, respectively [19]. Obesity has been reported to account for 30–40 % of kidney cancer cases in Europe and the United States; and is known to increase the risk of renal cell carcinoma in a dose–response fashion [12, 49]

In this study, residual spatial variation and resulting probabilities of exceedance for bladder and kidney cancer risk suggest that smoking is not the only factor contributing to the observed spatial patterns. This is because the proxy measures of smoking included in the analyses (i.e. social and material deprivation indices) did not change the spatial variations in risk or its magnitude. As well, the heterogeneity in bladder and kidney cancer risk observed in high risk areas was greater than what could be accounted by known spatial variations in smoking prevalence in Nova Scotia. Nonetheless, synergistic relationships between smoking and other un-measured risk factors cannot and should not be ruled out. This is especially important in Nova Scotia, a province known for its high prevalence of tobacco smoking [50], obesity [51] and where inorganic arsenic in drinking water was observed to be a major contributor to arsenic body burden in a study population [52]. Overall, the two spatial approaches used to model disease risk provided consistent and complementary results. Inclusion of a time-varying component in the spatially-continuous models permitted the determination of whether high average risk in a given location was sustained over time or changed over time; two different situations that could be derived from the same number of accumulated cases in an area over a set time period. As described by Abellan et al. [53], the epidemiologic interpretations of these two situations are important. In one scenario, spatial patterns are more likely to occur in a constant manner over time and hence could be induced by environmental or socio-demographic risk factors that act in a sustained manner. In the second scenario, the rate of case accumulation may be more temporally clustered with distinct variability, possibly reflecting emerging short-latency risk factors that would generate high excess cases in shorter time intervals or, alternatively, due to artificial or sudden variations associated with changes in disease coding or screening practices (see details in Abellan et al. [53]). Hence, it would not be unreasonable to suggest that the observed heterogeneity in the spatial distribution of high-risk areas for bladder and kidney cancer in both SW NS and CB, support a scenario in which risk factors act in a relatively sustained manner over time.

### Strengths and limitations

This study has important strengths. First, it is based on 30 years of cancer incidence data obtained from a population-based cancer registry adhering to registration standards of both the Canadian Cancer Registry and the North American Association of Central Cancer Registries. Those standards allow for consistency in disease coding over time and; ensure case ascertainment and completeness through a network of activities including automated and manual edit processes, record linkages and data audits. In addition, the systematic collection of spatial information at time of diagnosis enabled 100 % of cases in Cohort 1 and 95 % of cases in Cohort 2 to be successfully geo-referenced with a high degree of certainty, thus minimizing location misclassification (Cohort 1, ~ 85 % exact location; Cohort 2, ~ 50 %). Second, the two statistical methods used in this study accounted for spatial dependence (random effects) in risk estimates which reduce the likelihood of Type I error – declaring an area as having elevated risk when in fact its underlying true rate equals the background level [54]. Third, the exceedance probability rules, *P*_*i*_(10 %) > 0.8, *P(s;10 %)* > 0.8 and *P(s,t;10 %)* > 0.8, used here to classify spatial risk has high specificity even when data are sparse, further reducing the risk of false alarms, although perhaps increasing the likelihood of Type II error – declaring an area as having average risk when in fact its underlying true rate is elevated relative to background levels [54]. Fourth, the application of the local-EM algorithm treated risk as a continuously varying process in space and time and so was not constrained to be within arbitrary administrative boundaries which often change between census periods [52]. This allows for the integration and use of irregularly aggregated or point-location data within a single framework and minimizes loss of information. It presents a real advantage for the estimation of disease risk in small-area analyses or for rare diseases that requires the monitoring and accumulation of cases collected over a long time period as it maximizes statistical power and results in more meaningful inference [55]. As such, it is reasonable to suggest that applying the Local-EM framework improved the sensitivity of the study, offering a balance to the Community-level autoregressive model, a more conservative approach with generally lower sensitivity (see [54, 55]. Finally, modelling the spatio-temporal variation in risk with local-EM algorithm provided useful insights about the stability of the estimated spatial patterns of disease. It also produced predictions that were generally less spatially smooth, and as such, is a more sensitive tool for the detection of localized areas of elevated risk, which ultimately better informs health service planning, public health interventions and resource allocation.

Nonetheless, this study has limitations. First, location at time of diagnosis was used as a surrogate for the location where a person was thought to be exposed to factors which increased their risk of cancer. This is a common approach in the geographic analyses of many disease outcomes given the difficulty of obtaining a full history of residence and building estimates of lifetime exposure. The consequent exposure misclassification can result in less informative maps that impedes hypothesis generation or identification of environmentally or sociologically driven processes occurring over long time periods. Second, individual-level information on important risk factors such as smoking frequency and duration was not available as cancer registries do not routinely collect information unrelated to patient care. This study used neighbourhood social and material deprivation as a proxy for smoking prevalence. As a result, it is possible that maps of posterior means relative risks include some residual confounding due to smoking. Third, current algorithms for local-EM estimation do not allow for the inclusion of covariates. Fourth, the method is computationally intensive. Finally, although the local-EM analyses benefited from the inclusion of cases diagnosed over a longer time period, when reporting for the Cape Breton region, the number of cases was still quite low, which resulted in unstable results. This was particularly evident when determining optimal spatial and temporal bandwidths in females risk for which incidence counts was about 1.5 to 3 times lower than for males.

## Conclusion

Modeling the geographical distribution of disease within a population is essential to public health surveillance. It permits the quantification of the risk of disease relative to expected background levels, and the identification of unusually high and low risk areas which can guide health service planning, public health intervention and resource allocation. The current approach further permits the estimation of residual spatial dependence resulting from exposure to unmeasured risk variables, and as such, helps identify areas where other etiological factors may be at play. In this study, spatial analyses demonstrated evidence of spatial heterogeneity in the risk of both bladder and kidney cancers in Nova Scotia. The temporal component of the spatially-continuous approach permitted the determination of the relative time scales of high average risk in a given area and hence provided an understanding of the stability of the spatial patterns of the estimated risk; and the generation of hypotheses about the nature of possible exposure. Based on this information, we suggest that the excess bladder and kidney cancer risk for both male and potentially, female in south-western NS may be driven by exposure to unknown risk factors that act in a sustained manner over time. Further research may uncover the nature of these factors and lead to future opportunities for disease prevention.

The findings from this study warrant further investigation in three main areas. First, further work is required in the area of exposure modeling in order to elucidate the potential factors driving the observed patterns of variations in the risk of UTC in NS. Second, they highlight the need for the development of local-EM methods that incorporate individual- and neighborhood-level covariates. Finally, they reaffirm the need for the establishment of a public health platform that would enable the collection of individual- and/or neighborhood level information relating to disease causing-risk factors, such as behavioural, occupational and environmental factors. Such information permits more accurate quantification and understanding of disease risk.

## Additional files

Additional file 1:
**Analytical details [** 27 **,** 55 **,** 56**].** (DOC 42 kb) Additional file 2:
**Spatial cross-validation scores for the selection of optimal bandwidths.** (PNG 152 kb) Additional file 3:
**Temporal cross-validation scores for the selection of optimal bandwidths.** (PNG 109 kb)

## Abbreviations

BYM: Besag York and Mollie
CB: Cape Breton
Local-EM: local expectation maximization
NS: Nova Scotia
SW: South-Western
UTC: urinary tract cancer
