# Supplementary material for: Small-area spatio-temporal analyses of bladder and kidney cancer risk in Nova Scotia, Canada
Source: BMC Public Health. 2016 Feb 19;16:175. doi: 10.1186/s12889-016-2767-9 (PMC4761137; doi:10.1186/s12889-016-2767-9)
Supplement: Additional file 1: — Analytical details [ 27 , 55 , 56]. (DOC 42 kb) [file 12889_2016_2767_MOESM1_ESM.doc]

**Community-level analysis –** The BYM model [13, 14] applied in this study has the case count *Y_i_* for each Community *i* modelled as Poisson distributed, with the expected count for Community *i* being the product of its relative risk *λ_i_* and an expected count *E_i_* derived from the age-specific incidence rates of NS applied to each Community's population composition. The model is log-linear, with the log of the relative risk *λ_i_* being the sum of an intercept *μ*, the contribution of covariates *X_i_β* and the spatial random effect *U_i_*. The model is written as

| *Y_i_*~ | Poisson(*E_i_* *λ_i_*) |
| --- | --- |
| log(*λ_i_* ) = | *μ* + *X_i_β* + *U_i_* |
| *(U_1_, U_2_ ... U_N_*)'~ | BYM(*σ^2^,τ^2^*) |

with the random effects *U_1_* to *U_N_* having a spatially dependent joint distribution where adjoining regions having a direct influence upon one another. The sum of the two BYM variance parameters *σ^2^*+ *τ^2^* governs the 'importance' of the effect (how close to or far from zero each *U_i_* is likely to be), with their ratio *σ^2^/τ^2^,* determining the smoothness or degree to which each *U_i_* is influenced by its neighbours. More specifically, each *U_i_* is the sum of an independent or unstructured random term and a spatially autoregressive (first order Gaussian Markov random field) component with variance parameters *σ^2^*and *τ^2^*, respectively.

Bayesian inference were applied for model fitting using Integrated Nested Laplace Approximations to calculate the posterior marginals [29]. Uninformative prior distributions were specified for the *μ* and *β* parameters with the *μ* having improper flat priors and the *β* being assigned Normal priors with mean zero and variance 1000 (N(0,1000)). The variance parameters *σ^2^* and *τ^2^* were given identical priors with 95% intervals between 0.025 and 1.0 which resulted in a fairly unrestrictive upper limit considering that log-relative risks of *U_i_* =-2 or *U_i_* =2 (plus or minus two standard deviations) correspond to relative risks exp(*U_i_* ) of 0.135 or 7.4, respectively.

**Spatially-continuous analysis**– In applying the Local-EM kernel smoothing algorithm the algorithms for the locations of cancer cases are random Poisson process with an intensity surface at each location in space *s* and time *t* being the product of a `offset' surface *O*(*s,t*) derived from the population at risk and a smoothly varying relative risk *λ*(*s,t*). A local-likelihood algorithm is similar to a kernel smoother, with a kernel function *K*(*s-X_i_, t-T_i_*) specifying the weight to assign to a case *i* located at (*X_i_,T_i_*) for the purpose of estimating risk *λ*(*s,t)* at *s* and *t*. The local-EM algorithm deals with unobserved (or censored) locations (*X_i_,T_i_*) by having an estimate *λ*(*s,t*) being the maximum of an expected likelihood subject to the constraint that (*X_i_,T_i_*) be located within the case's known census or postal region.

The local-EM algorithm does not impute a single location for each case. Rather, the estimated risk surface averages out all the possible locations at which each case could be located. The risk surface is sensitive to the bandwidth of the smoothing kernel used, with wider bandwidth giving smoother and flatter risk surfaces. Shorter bandwidths return rougher surfaces and result from data inherently more heterogeneous where close neighbours— in space or time, have the greatest influence on risk estimation. The bandwidth of the kernel functions (one each in time and in space) were chosen by cross-validation (see Appendix B-C), where data were systematically excluded from model fitting and the optimal bandwidths being those that are best able to predict the excluded data.

Finally, the *O*(*s*,*t*) offset surface was calculated from: the population density for each age-sex group at the relevant location and during the most proximate census of population; an age and sex specific rate obtained from a reference population; and a yearly-varying relative risk term ensuring the observed count in each year and the total number of cases expected in that year are equal. A relative risk of λ(*s*,*t*)=1 everywhere would indicate *O*(*s*,*t*) is an accurate quantification of the distribution of incident cases, whereas λ(*s*,*t*) above or below 1 indicate a surplus or deficit of cancer cases respectively.
