# Supplementary figures and images for: Small-area spatio-temporal analyses of bladder and kidney cancer risk in Nova Scotia, Canada
Source: BMC Public Health. 2016 Feb 19;16:175. doi: 10.1186/s12889-016-2767-9 (PMC4761137; doi:10.1186/s12889-016-2767-9)

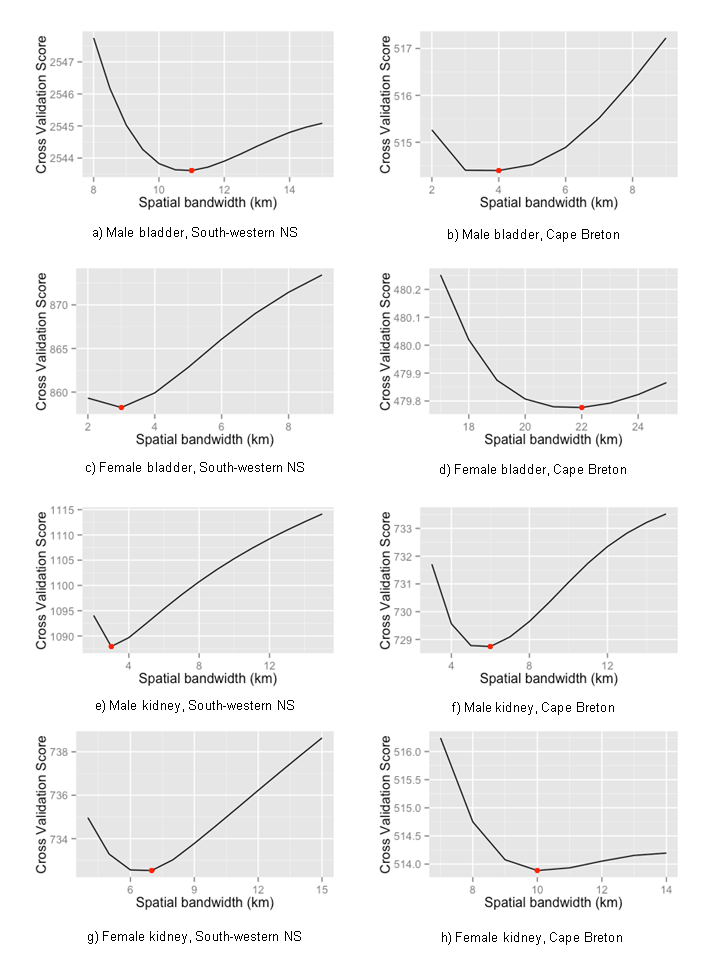

Supplement: Additional file 2: — Spatial cross-validation scores for the selection of optimal bandwidths. (PNG 152 kb) [file 12889_2016_2767_MOESM2_ESM.png]

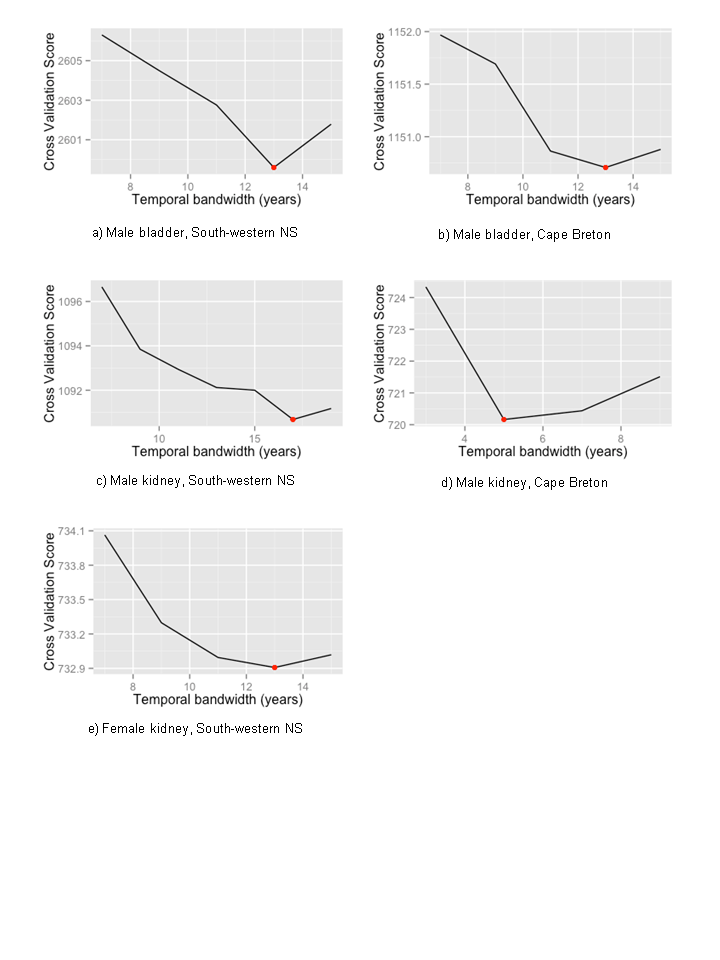

Supplement: Additional file 3: — Temporal cross-validation scores for the selection of optimal bandwidths. (PNG 109 kb) [file 12889_2016_2767_MOESM3_ESM.png]
